# ‘I JUST WANT TO RUN’: how recreational runners perceive and deal with injuries

**DOI:** 10.1136/bmjsem-2021-001117

**Published:** 2021-09-27

**Authors:** Evert Verhagen, Marit Warsen, Caroline Silveira Bolling

**Affiliations:** Amsterdam Collaboration on Health & Safety in Sports, Department of Public and Occupational Health, Amsterdam Movement Sciences, Amsterdam UMC, University Medical Centers – Vrije Universiteit Amsterdam, Amsterdam, The Netherlands

**Keywords:** running, injury, prevention

## Abstract

Running injuries impact the health gains achieved through running and are linked to drop-out from this otherwise healthy activity. The need for effective prevention is apparent, however, implementation of preventive measures implies a change in runners’ behaviour. This exploratory qualitative study aimed to explore Dutch recreational runners’ perception on injuries, injury occurrence and prevention. An interpretative paradigm underpins this study. We conducted 12 individual semistructured interviews with male (n=6) and female runners (n=6). Through a constant comparative data analysis, we developed a conceptual model to illustrate the final product of the analysis and represent the main themes’ connection. We present a framework that describes the pathway from load to injury and the self-regulatory process controlling this pathway. Runners mentioned that pain is not necessarily an injury, and they usually continue running. Once complaints become unmanageable and limit the runner’s ability to participate, an injury was perceived. Based on our outcomes, we recommend that preventive strategies focus on the self-regulation by which runners manage their complaints and injuries—providing information, advice and programmes that support the runner to make well-informed, effective decisions.

**Key messages**

**What is already known**

- Running, as a recreation physical activity, has positive health effects but is also characterised by a high number of injuries.
- There is evidence showing that it is possible to prevent injuries in runners. However, implementation of this evidence into the practice of running is challenging.
- Previous descriptive studies described the opinions and beliefs of runners regarding injuries and their prevention.

**What are the new findings**

- Runners perceive complaints as a normal part of their running practice. However, when injuries hamper their participation and autonomy to run, they considered themselves injured.
- Injury prevention is not a conscious decision for recreational runners but a tentative to control and influence the injury through a self-regulation process.
- We recommend that preventive strategies focus on the self-regulation process and facilitate self-efficacy and empowerment to help runners manage complaints and injuries.

## Introduction

Running is a very popular activity, enjoyed by many around the globe. Without argue running has great positive effects on the individual’s physical and mental health.^1^ Running, however, is also characterised by a high number of injuries.^2–6^ These injuries impact the health gains achieved through running and are even linked to drop-out from this otherwise healthy activity.^7–9^

There is evidence showing that it is possible to prevent injuries in runners.^10–12^ Most of the available interventions aim to change individual risk factors (ie, strength, stability, load) to reduce the risk of injury.^2 12^ For these interventions to be effective, the runners need to adhere to the provided advice. However, as with most injury prevention programmes, implementing this evidence into the practice of running is challenging. The implementation of preventive measures implies a change or modification of an athlete’s behaviour.^13–15^ When introducing preventive measures and evaluating the effect of such measures, it is necessary to know the determinants of such preventive behaviours.

Previous studies described the opinions and beliefs of runners regarding injuries and their prevention.^8 16–20^ However, these insights stem from quantitative surveys. If we want to know why runners behave as they do and how they deal with injury and injury risk, qualitative research should be used to understand the runners’ perspectives.^21^ Consequently, our study aimed to explore, through a qualitative approach, Dutch recreational runners’ perspectives regarding injuries, their care and their prevention.

## Methods

### Design

This is an exploratory qualitative study in which an inductive analysis developed the understanding of meanings and concepts around running-related injury based on the participants’ voice. An interpretative paradigm underpins this study.

### Participant selection

This study had a convenience sample composed of recreational runners in The Netherlands. The participants were recruited in two local Dutch running clubs in Dordrecht (n=3) and Eindhoven (n=9). One of the researchers (EV) had personal contacts at these clubs who communicated a call for participation. Initial participants provided contacts for further potential participants using a respondent-driven sampling method. We estimate that 31 runners received our call for participation, of which 13 responded positively. Participants were informed of the study’s background and goals, after which they provided verbal informed consent. Reporting followed the recommendations based on the Consolidated Criteria for Reporting Qualitative Research^22^ (online supplemental appendix 1).

10.1136/bmjsem-2021-001117.supp1 Supplementary data

### Reflexivity

All authors are trained and experienced in conducting qualitative research with athletes. EV is a sports scientist and epidemiologist, experienced runner and running coach. CSB is a sports physical therapist, postdoctoral researcher and experienced runner. MW has a bachelor’s degree in health sciences and has no running experience. The variety of views and backgrounds represented by the authors supports the neutrality of our findings.

### Data collection

According to participants’ availability, the principal author (EV) conducted all the individual semistructured interviews between November 2019 and May 2020. Interviews were conducted in order of participant acceptance. The interview structure covered the topics: running experience and motivation, injury definition, injury experiences, perceived risk factors and injury prevention strategies (online supplemental appendix 2). After 11 interviews, the main ideas and concepts repeated themselves. To ensure that data saturation was achieved, we conducted one more interview, and no additional information emerged.^23^ According to participants' preference, four interviews were done face to face at the running club and eight by phone. Interviews were conducted in Dutch (n=11) and English (n=1).

10.1136/bmjsem-2021-001117.supp2 Supplementary data

### Data analysis

Interviews were audiorecorded and transcribed verbatim. Transcripts were not returned to participants for comment or correction. The analysis process used the original transcripts in Dutch and English. The presented quotes of Dutch interviews were translated into English by MW and reviewed by EV.

**Figure 1:**
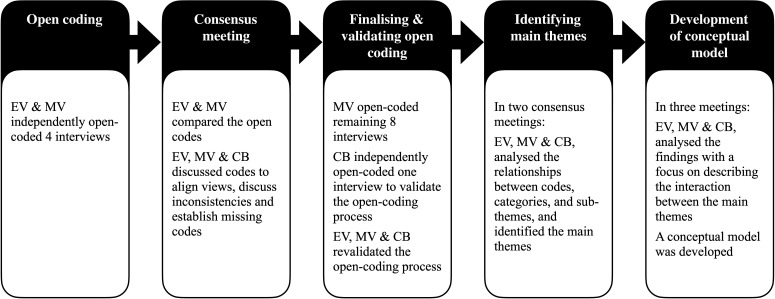
Schematic presentation of the data analysis process.

We employed constant comparative data analysis.^24 25^ First, in ATLAS.ti (V.9), four interviews were open coded independently by EV and MW. Both are Dutch native speakers. Subsequently, EV and MW discussed codes and their impressions with CSB, who was not familiar with the interviews’ content. CSB also independently coded one interview to test assumptions and coherence in interpretation of the coding process. After consensus on the main codes, the remaining interviews (n=8) were coded by MW. In two meetings, all authors analysed and discussed the relationships between codes, categories and subthemes to identify the main themes. After that, we developed a conceptual model to illustrate the final product of the analysis and represent the main themes' connection. A schematic representation of this process is presented in figure 1.

## Results

### Demographics

Our sample consisted of six male and six female recreational runners (table 1). The average age was 43.1 years (SD 9.2), and the average running experience was 10, 5 years (SD 7.5). The interviews’ average duration was 14.8 min (SD 3.4), ranging from 9 min to 19 min.

**Table 1:** Main demographics of the study sample

Participant	Sex	Age range (years)	Running experience (years)
#1	Male	50–55	29
#2	Female	30–35	9
#3	Female	45–50	8
#4	Female	40–45	11
#5	Male	35–40	3
#6	Female	55–60	12
#7	Male	35–40	2
#8	Male	55–60	16
#9	Female	40–45	10
#10	Male	25–30	1
#11	Male	40–45	12
#12	Female	40–45	14

### Why do I run?!

Participants report different motivations to run (table 2). Almost all participants state to run to achieve physical health benefits. Other positive aspects of running that were mentioned revolve around the social context and the distraction from everyday hassles. Only a few participants state to run with a motivation to improve their performance.

**Table 2:** Themes, subcodes and exemplary quotes on reasons to participate

Main theme	Subcode	Exemplary quote
Motivation	Health benefits	Runner 11: ‘I just want to stay in good shape and keep my fitness level up.’
		Runner 10: ‘For me, health is the most important factor. I have diabetes, so that is really a prime reason for me. I notice that my insulin sensitivity is terribly dependent on the amount of activity I have in a day.’
	Social contacts	Runner 6: ‘Yes, I enjoy it, the social aspect. I started to get to know people in our neighbourhood.’ Runner 2: ‘I really am a social runner. It motivates me to train with a fixed group of people at a fixed time, and I like that more than running on a grey Thursday evening by myself.’
	Performance	Runner 3: ‘I want to improve myself. I don't have to. I don't do it for someone else. I want to be faster myself. I like that personal best.’
		Runner 9: ‘I am more challenging myself and being, you know, in a permanent challenge with my own performance.’
	Distraction	Runner 9: ‘I am working mostly mentally, being in the IT industry. So, running is also helpful to clean up my head after work.’
		Runner 10: ‘I de-stress while running, and especially now that we have two children, it is really nice to just close the door behind you every now and then.’

### Too much, too fast, too long

Participants mentioned overloading during the interviews, leading to physical complaints (table 3). They described this overloading as: ‘running too much, too fast or too long’. The lack of preparation, rest and general fatigue were described as contributing factors to overload.

**Table 3:** Themes, subcodes and exemplary quotes on the onset and care for complaints

Main theme	Subcode	Exemplary quote
Overloading	No proper build-up	Runner 2: ‘If you suddenly scale up your intervals, training sessions, or distances because you want to train from a half marathon to a full marathon. Those are the risky points.’
	Lack of rest	Runner 8: ‘You know, when I was running six days per week doing an average per day of, I think, around 11 kilometres and still pushing on a speed of around 40 to 44 min then after a period of time, after a few months without having a proper rest you know, these things accumulate, and the muscles became much more fragile eventually.’
	General fatigue	Runner 5: ‘If I've had a day where I've been massaging [job] really intensively, and I would indeed have to train on Monday, and I vaguely feel that I'm thinking 'ooh'. Then I think, maybe it would be smarter not to go tonight. It is not so much that I feel an injury or something, but it is more like general fatigue or just not feeling like it.’
		Runner 10: ‘Yes, fatigue can really be a factor. If you are really tired, running can help against that fatigue, but then it is smarter to run 5 kilometres instead of 15.’
Complaints	Small pains	Runner 6: ‘As long as it is not too much, just a pain, then it is not so bad. Then I think that it will go away, and often it will.’
	Ability to continue	Runner 11: ‘When running, I have times when I have pain at my ankle or something, but I just walk through it.’
		Runner 5: ‘If I really seriously couldn't run anymore, that would really, yes, be the point for me to say, well, yes, that is really an injury. But look, if I have a pain here or there, I will not immediately stop and see that as an injury.’
Self-regulation	Adjust load	Runner 2: ‘If you have had a tough week, it does not mean that you should not go for a run even though you feel tired. You may say: well, I will take it a little less far or a bit easier.’
Influencing factors	Competitive drive/performance goals	Runner 12: ‘There are always runners who think 'hmm, John always runs slower than me, and now John is running faster than me, but I don't want that to happen. So, I’m still going to push to come in well before John.’ You really see that happen every training. I can almost blindly point out to whom it is going to happen.’
		Runner 5: ‘I am not saying that that is the bulk, certainly not, but yes, people who are really fanatic and really train to improve their performance. They eventually will start to feel a pain somewhere or be injured or get injured.’
	Race planning/training schedule	Runner 2: ‘Because Valencia is my only marathon this year, I think it’s a shame. I will go to Spain for a whole weekend. I am training for three months. I'm not going to have this plan ruined.’
	Everyday life	Runner 12: ‘Besides running, I try, in any case, to be very physically active. I have a physically strenuous job, so I am busy all day. I am not someone who sits still. I also have to walk the dog regularly, so yes, I still have an active day besides running. I think that also plays a role.’
	Social control	Runner 12: ‘I am running with a group, and I do not want to let that group down.’
		Runner 6: ‘Running in a group gives you some pressure, like the feeling. you have an appointment, and you have to go.’

If they have pain but can still run, participants considered that they have a complaint or ‘just a small pain’ and not an injury. Some runners reported that these complaints are a ‘normality’ of a runners’ life. The participants described that they manage their complaints by adjusting the distance, speed, frequency or duration of their running activities. This self-regulation process was mentioned to be influenced by their competition schedule, performance goals, competitive drive or daily personal life.

### When I can’t make my own choices, I am injured

When no improvement was experienced through the adjustments made or by an aggravation of complaints, the participants considered themselves injured. An injury was described by participants when complaints overtake their autonomy on their running activities, their ability to run at the level they want or run at all (table 4). The runners’ training level and experience influence this path from complaint to injury. For example, one participant (runner #5) mentioned that when he started with running, complaints were in some way expected, and the presence of pains and aches were expected to reduce with the progression of training. However, once trained, complaints are taken more seriously and are earlier considered to be an injury.

**Table 4:** Themes, subcodes and exemplary quotes on definition and care of injury

Main theme	Subcode	Exemplary quote
Injury	Loss of autonomy	Runner 10: ‘If I can't do what I need or what I normally can do.’
		Runner 12: ‘An injury for me is when I have pain, complaints or some dysfunction that hinders the training that I want to do and when I cannot do my training in an adapted form.’
		Runner 4: ‘An injury to me is something that is a nuisance while running, or before or after. Something that hinders me in my normal movement, in what I normally do. That doesn't have to be something with your legs, but that can also be something in your arms or your shoulder, something that hinders in the training that you normally do or everyday life.’
	Unable to run	Runner 6: ‘Yes, if you really can't walk anymore. That you really need to rest for a few weeks and then slowly start building up again. I would say that’s an injury.’
Influencing factors	Training level	Runner 8: ‘At first, I thought it was really a build-up phase because the first time I ran, I was already suffering from my hips, so you can imagine. So I imagine myself, if you go to a gym, and I never go to a gym, but if I go to a gym and I bench press in one go, and I have arm pain the next day, I think that’s more something like overtraining, I don't call that an injury.’
		Runner 6: ‘I think my muscles were still in the build-up phase and especially those, yeah, I just think still need some development.’
	Runner’s experience	Runner 1: ‘With the first injury, you think it will run loose, but now for me, this is something where I worry quicker. My body is also getting older. Maybe I ask too much of my body, even though I can do it all between my ears.’
		Runner 5: ‘I think it’s very personal, of course. I think a recreational runner might be more likely to say yes, listen, this isn't worth it to me. While someone who really trains hard for something will then think yes, this is not fitting my planning.’
Self-regulation	Absolute rest	Runner 12: ‘When my ankle didn't want to go, I just rested for a few days, and that was it. Then I could continue.’
	Medical care	Runner 1: ‘I had muscle pain in my buttock the week before a race, and that did not go away. So then I went to the physiotherapist, and he said, well, buddy, I think this is not your buttock because after 1.5 weeks muscle pain should be gone. So I’m under treatment now.’
		Runner 8: ‘I went to a foot therapist, and I had orthotics measured there. That wasn't enough. Somehow that didn't work well. Then I put in an in-sole just from another shoe with a high instep, and now it’s over, so to speak.’

The participants described two main approaches to deal with their injury. Initially, most participants mentioned taking absolute rest and not run for a while. Some participants mentioned that they would resume their running activities after this resting period even though they still had complaints. Not all participants stated to seek professional care for their injury, and they would only seek further care if the injury ‘demanded’ so.

### Preventive behaviours

Runners did not report a conscious will to prevent injuries. Based on our analysis, self-regulation is the main process by which runners deal with complaints and injury (table 5). Participants reported that this process is driven by their own experience, the information they seek actively (mostly online), or information they receive through professionals and peers. The latter is reported to happen primarily passively, for example, through coaches’ actions or conversations with fellow runners. Participants could not easily describe their preventive efforts but stated to buy new shoes regularly, follow a tailored training schedule or perform core stability exercises. Runners did not specifically report that these actions were taken as conscious strategies to reduce their risk of injury.

**Table 5:** Themes, subcodes and exemplary quotes on self-regulation

Main theme	Subcode	Exemplary quote
Experience and knowledge	Own experience	Runner 10: ‘I'm a little older now and hopefully wiser, and I hope those mistakes that I used to make I at least make less now.
		Runner 5: ‘I think it’s a little bit of both, a little bit of just experience, a little bit of living up to your own feelings and also a little bit of, yeah, some knowledge that you just know. I just know that if you overwork your body, you overload it.’
		Runner 12: ‘I actually developed over the course of those 14 years that I know very well where my limits lie. This is, of course, created by experience, if you go for a stroll through a city one day and you go for a long endurance run the next day, and you notice that you get tired much faster and that it bothers you the rest of the day then I think, well, I don't like that, so next time I'm going to plan it a little differently so that it bothers me less. Or maybe I'll skip the training because the combination is not going to work.’
	Online resources	Runner 1: ‘I first consulted Doctor Google and to find out about supplements you need for muscle building or strengthening of joints and bones and so on. So yes, I am actively looking for that.’
		Runner 8: ‘When I started running, I had problems with my hip, and then I watched a lot of videos on YouTube about where it could come from and how you should run. So I mainly focused on running techniques, how should I run, what should my posture be. That helps me a lot during training.’
		Runner 6: ‘Proper trainer schedules can also be found on the internet if you would like.’
	Peer opinion	Runner 10: ‘We do have now at our running group a number of people who know very well what they're doing as trainers. We have a physiotherapist as a trainer. We have a professor in exercise science or something. But anyway, those are the people who really do drill us in a proper way to learn habits that make us less likely to get injured.’
		Runner 5: ‘You also run with a group of runners who all keep up their knowledge. Some more than others, but I do think that we hear quite a lot. during a training.’
		Runner 3: ‘I follow him the trainer because he has the experience, and so far, I have really never had an injury or muscle pain or anything.’
	Expert advice	Runner 1: I think that it is an obligation of a trainer to make runners aware that the body also has its limits even though it feels so good now, and that actually sometimes rest is the best training.’ Runner 10: ‘I try to pass it on to the other runners. With some regularity runners join us, and they are all very motivated. I don't want to slow them down in their enthusiasm, but I do want to say, guys, be careful and just take your time.
		Runner 4: ‘Yes, I am always right on time with my consultation at the physiotherapist, and they also know that I should not go over it six times. I just have to get started briefly and powerfully.’

### The injury pathway

**Figure 2:**
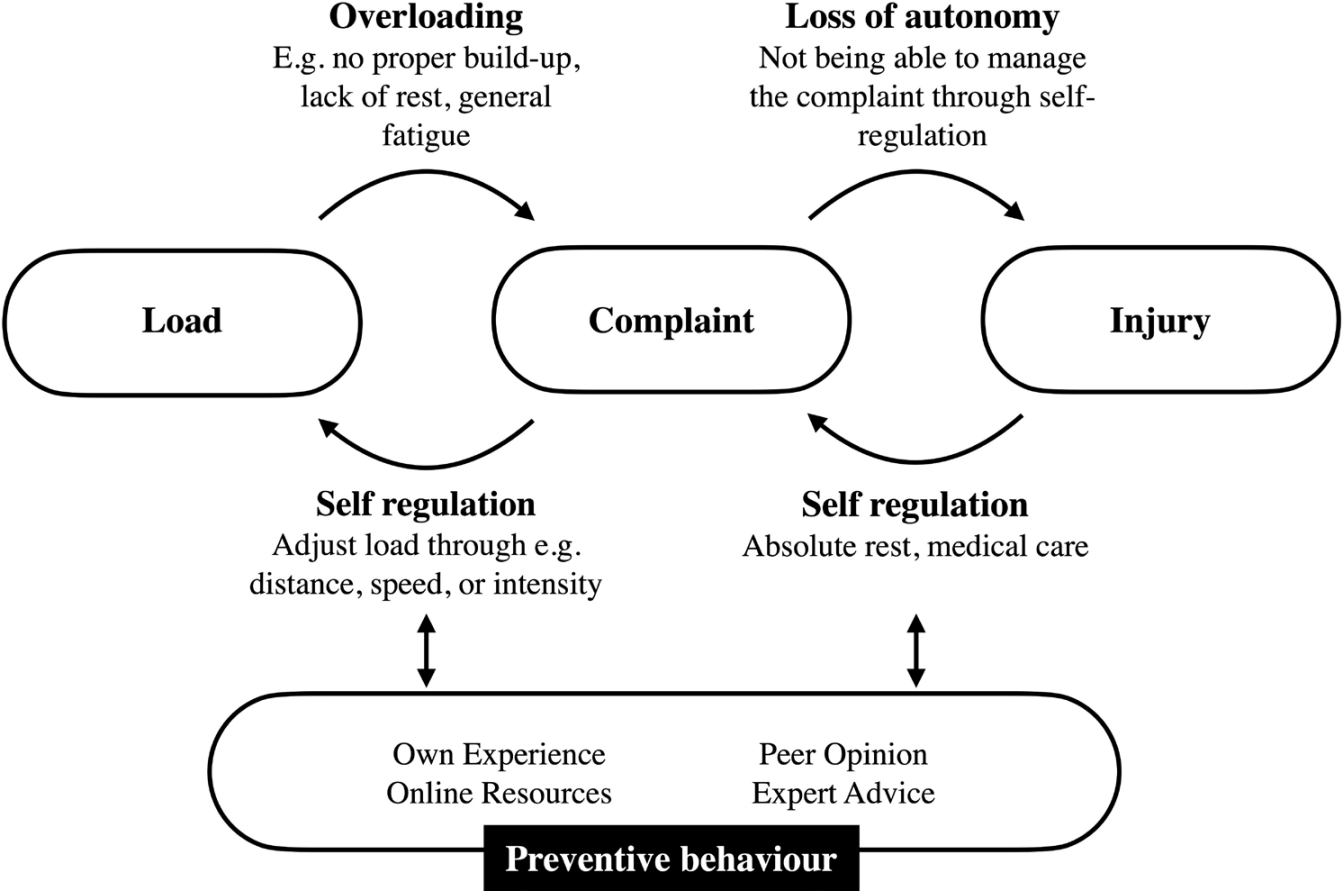
The injury pathway as experienced by recreational runners.

Runners described injuries as the outcome of a process (figure 2). Running is understood to be a physical load to which the body reacts and adapts. When this load is disproportionally balanced—generally coined ‘overloading’—pains and aches (complaints) develop. Through self-regulation of load, complaints can be either managed or resolved. If a complaint is not controlled successfully, the runner loses autonomy, and the complaint dictates the course of action. This is the moment the runner considers to be injured. Self-regulation—usually by taking absolute rest—may allow the runner to get back participating with a manageable complaint. In some cases, medical advice and care are sought. The self-regulation process is supported by knowledge and expertise gained through experience and information derived passively and actively through peer opinions, online resources and expert advice. As a result, the runner develops preventive behaviours.

## Discussion

This study provides an insight into how recreational runners perceive and deal with running-related injuries. We derived a framework that describes the pathway from load to injury and the self-regulatory process controlling this pathway.

### Aches and pains are part of the game

Runners mentioned that pain is not necessarily an injury, and with small pains, they usually continue running. This finding is supported by other literature that found that runners keep running with pain.^8 17 26–28^ Once their complaints become unmanageable and limit the runner’s ability to participate, an injury was perceived. Similarly, a previous qualitative study with elite athletes found that athletes defined an injury not guided by pain but as a complaint that hampers their performance.^29^

In this way, we can argue that finding tools to monitor and support the management of runners’ complaints could minimise the impact of potential injuries for recreational runners. A previous trial by Hespanhol *et al*^11^ did just that. They provided runners preventive feedback and advice once complaints were registered. Compared with a control group, who only received general advice, the intervention participants showed increased adherence to the advice given and lower injury rates.

This finding also implies that we must consider the injury definitions employed in our studies. The recent IOC consensus on recording and reporting epidemiological injury data describes an injury as tissue damage or other deviation of normal physical function.^30^ This is a very inclusive definition that encompasses different components of our framework. However, most studies employ an operational definition with a more limited scope, like time-loss and a need for medical care. Such narrow definitions only paint part of a bigger picture. Our findings support the use of a broader definition, such as recommended by the IOC.

### From load to a complaint

The process of overloading, as described by recreational runners, is also described in the literature as a factor that leads to injury.^31 32^ This was also mentioned by runners in a previous larger survey^18^ and found in prospective studies.^17 33^ Overloading, in general, can occur due to a combination of wanting to do too much, too fast and for too long without proper rest. Reasons for this are stated to be a joy of running or to achieve a specific goal, and, therefore, one is unwilling to stop. This phenomenon has also been described by León-Guereño *et al*,^34^ who found that intrinsic motivation was associated with a higher incidence of injury.

It was interesting to find that, where it concerned overloading, participating runners mentioned only load related factors. Overloading, however, can also be caused by an insufficient or reduced load capacity.^35^ For endurance sports, inclusive of running, for instance, a lack of sleep and psychological factors have been linked to insufficient recovery and increased injury risk.^36 37^ However, such factors do not seem to be in the imaginary of recreational runners to contribute to overloading.

As reported by previous surveys,^16 28 38^ our interviewed runners have their relationships with running. Some seek to partake for health benefits, others describe the social aspect, and some state the performance aspect as a main motivator to participate. Based on their primary motivation to participate, runners have set their own goals and personally challenge themselves. Achieving individual goals or challenges is an important factor influencing the risk of overloading, self-regulation methods, and a feeling of loss of autonomy. In this way, it is important to understand the runners’ motivation to help them manage the risk of injury.

### The autonomous state of recreational runners: self-regulation

Autonomy was an underlying concept in our study. Runners want to determine, based on their feelings, when to alter their training programme, take additional or prolonged rest or take other measures. Although autonomy is not mentioned frequently in the literature, our findings are corroborated by previous quantitative studies^17 19 20 28^ and a previous qualitative study in competitive runners.^39^ These findings imply that runners deal with complaints through self-regulation. Self-regulation is a learning process through which the information from peers, experts, (online) media and previous—positive and negative—experiences improve the runner’s skill to deal with complaints and injuries. This process was previously also found by Bolling *et al* for elite athletes.^29 40^ The main difference lies therein that in the elite context, athletes, coaches and staff work together to regulate the load and that in a recreational setting, an athlete acts autonomously.

The idea of autonomy is also related to the concept of ‘empowerment’. Based on the WHO definition, empowerment is ‘a process through which people gain greater control over decisions and actions affecting their health’. It is easy to draw a parallel of this concept to our participating runners who want to control factors related to their complaints and injuries. Empowering athletes is a recent topic in sports medicine, mostly in the return-to-sports literature.^41^ For injury prevention strategies, most of the interventions are made for athletes but not with athletes and have no focus on developing self-efficacy and empowerment. Our findings show that the runner is seeking autonomy and self-regulation. Therefore, efforts to reduce the risk of running injuries should also allow runners to practice their self-efficacy.

### Prevention of injuries?

We found that our runners could not easily describe the preventive efforts they take, and it seems that runners are unconsciously engaged in injury prevention. Participants stated to buy new shoes regularly, follow a tailored training schedule or perform core stability exercises, but these strategies were not systematic and linked to injury prevention. Previous studies explored the beliefs and opinions of runners on the causes and prevention of injuries, stating similar factors like shoes, stretching and load management.^18–20 38 39^ Measures which runners reason as effective, but for which most no sound evidence is available.

An interesting finding is that preventive behaviours are influenced by previous experiences, acquired knowledge and advice from peers. Runners gather these in an ‘inductive’ way through different channels, both actively and passively. Sometimes they are looking for information on the internet or ask their peers. Other times, they get their information through advice and feedback given by experts. Education is, in general, a perpetual topic in sports injury prevention which highlights the importance of knowledge and information.^42^ Our study found that it is not only about what runners learn, but mostly how, from whom and through which channels. To our knowledge, this is a novel finding among recreational runners and provides important insights for the implementation of preventive advice and interventions for this population.

### Limitations and strengths

Regarding transferability, when interpreting the findings of our study, one should consider that our sample consisted of a broad cross-section of Dutch recreational runners. As reported in quantitative studies, injury risk and injury risk factors vary by demographics, for example, age, gender, experience, motivation, etc. We must, therefore, consider that such factors also influence the injury process we describe. In our interviews, we did notice some differences in responses between participants, confirming this consideration. Consequently, our findings apply to a general recreational running population only and considerations for specific recreational runners should be a topic for further research. Further, our sample was restricted to only two running clubs from the Netherlands, and all runners were running in a group. We acknowledge that ‘solo’ recreational runners or elite runners may have different contexts.

We applied measures to improve the trustworthiness of our study. The analysis process with independent coders and the different backgrounds of these coders enhanced the credibility of our outcomes. We should make note, however, of the potential that the coders’ background influenced the analyses. The running and academic experience—focused on injury prevention—of both EV and CSB, could have unconsciously provided interpretations to participants’ responses in the coding process. To avoid any influence of these backgrounds and previous experiences, the coding was conducted by MW, who had no running nor scientific history related to the topic of this study. The multiple meetings and discussions to validate the analysis and the connections made with previous quantitative literature enhance confirmability.

### Practical implications

The outcomes of our study provide an understanding of recreational runners’ perception on injuries, injury occurrence and prevention. We present a framework that describes the pathway from load to injury and the self-regulatory process controlling this pathway. The development of an injury is a process, and to avoid the onset of injury, we should look for ways to act on this process. Our framework provides tangible opportunities to do so.

Based on our outcomes, we recommend that preventive strategies focus on the self-regulation by which runners manage their complaints and injuries—providing information, advice and programmes that support the runner to make well-informed, effective decisions. In doing so, we should consider that runners have different motivations to participate, affecting their choices in the self-regulating process. We should also be aware that recreational runners are also unconsciously exposed to injury prevention advice and practices, for instance, through peers and experts. These channels may provide important, not yet used, conduits to bring preventive evidence to the recreational runner.

## Data Availability

Data are available on reasonable request. The dataset analyzed during the current study is available from the corresponding author on reasonable request.
