# Supplementary material for: ‘I JUST WANT TO RUN’: how recreational runners perceive and deal with injuries
Source: BMJ Open Sport Exerc Med. 2021 Sep 27;7(3):e001117. doi: 10.1136/bmjsem-2021-001117 (PMC8477337; doi:10.1136/bmjsem-2021-001117)
Supplement: Supplementary data [file bmjsem-2021-001117supp001.pdf]

Appendix 1 Consolidated criteria for reporting qualitative studies (COREQ)

Developed from: Tong A, Sainsbury P, Craig J. Consolidated criteria for reporting qualitative research (COREQ): a 32-item checklist for interviews and focus groups. *International Journal for Quality in Health Care*. 2007. Volume 19, Number 6: pp. 349 – 357

No. Item	Guide questions/description	Explanation	Reported on Page #
**Domain 1: Research team and reflexivity**			
Personal Characteristics			
1. Interviewer/facilitator	Which author/s conducted the interview or focus group?	All the individual semi-structured interviews were conducted by Evert Verhagen	6
2. Credentials	What were the researcher's credentials? E.g. PhD, MD	Evert Verhagen, PhD<br>Caroline Bolling, PhD<br>Marit Warsen, BSc	-
3. Occupation	What was their occupation at the time of the study?	Evert Verhagen, Professor<br>Caroline Bolling, Researcher<br>Marit Warsen, Master Student	-
4. Gender	Was the researcher male or female?	The interviewer is male, the other authors are female	-
5. Experience and training	What experience or training did the researcher have?	Evert Verhagen has a masters degree in epidemiology, a PhD in Medicine, and is a trained qualitative researcher.<br>Caroline Bolling has a masters degree in physiotherapy, a PhD in Medicine, and is an experienced qualitative researcher.<br>Marit Warsen has a bachelor degree in Health Sciences and experience with qualitative research.	5
Relationship with participants			

6. Relationship established	Was a relationship established prior to study commencement?	No	5
7. Participant knowledge of the interviewer	What did the participants know about the researcher? E.g. personal goals, reasons for doing the research	Participants were informed on the background and goals of the study before the interview	5
8. Interviewer characteristics	What characteristics were reported about the interviewer/facilitator? e.g. Bias, assumptions, reasons and interests in the research topic	EV has a background as a runner and running coach, is an epidemiologist in sports medicine, and has experience with qualitative research	5
**Domain 2: study design**			
Theoretical framework			
9. Methodological orientation and Theory	What methodological orientation was stated to underpin the study? e.g. grounded theory, discourse analysis, ethnography, phenomenology, content analysis	An interpretative paradigm underpins this study.	5
Participant selection			
10. Sampling	How were participants selected? e.g. purposive, convenience, consecutive, snowball	We used a respondent-driven sampling method	5
11. Method of approach	How were participants approached? e.g. face-to-face, telephone, mail, email	One of the researchers (EV) had personal contacts at these clubs through which our call for participation was communicated	5
12. Sample size	How many participants were in the study?	12 participants in total	5
13. Non-participation	How many people refused to participate or dropped out? Reasons?	18 participants who received our invitation did not respond. Reasons for non-response are unknown.	8

Setting			
14. Setting of data collection	Where was the data collected? e.g. home, clinic, workplace	Data was either collected face -to-face at the sports club (n=4) or by phone (n=8).	6
15. Presence of non-participants	Was anyone else present besides the participants and researchers?	All interviews were conducted without anyone else present.	6
16. Description of sample	What are the important characteristics of the sample? e.g. demographic data, date	These are described in Table 2	8
Data collection			
17. Interview guide	Were questions, prompts, guides provided by the authors? Was it pilot tested?	This is outlined in Table 1	6
18. Repeat interviews	Were repeat interviews carried out? If yes, how many?	No	-
19. Audio/visual recording	Did the research use audio or visual recording to collect the data?	All interviews were audio-recorded	7
20. Field notes	Were field notes made during and/or after the interview or focus group?	Yes, field notes were made during data collection.	-
21. Duration	What was the duration of the interviews or focus group?	The average duration of the interviews was 14,8 minutes (SD 3,4 minutes), ranging from 9 minutes to 19 minutes.	8
22. Data saturation	Was data saturation discussed?	Data satiation was reached after 11 interviews and confirmed by one additional interview.	6
23. Transcripts returned	Were transcripts returned to participants for comment and/or correction?	No	7

Domain 3: analysis and findings			
Data analysis			
24. Number of data coders	How many data coders coded the data?	Two coders independently coded 4 interviews. After consensus one coder coded the remaining interviews.	7
25. Description of the coding tree	Did authors provide a description of the coding tree?	Yes, we provide a conceptual map as a result from the data analysis	8
26. Derivation of themes	Were themes identified in advance or derived from the data?	Themes were derived from the data	7
27. Software	What software, if applicable, was used to manage the data?	Atlas.TI	7
28. Participant checking	Did participants provide feedback on the findings?	Participants did not provide feedback	-
Reporting			
29. Quotations presented	Were participant quotations presented to illustrate the themes/findings? Was each quotation identified? e.g. participant number	Yes, in the results in Tables	10-19
30. Data and findings consistent	Was there consistency between the data presented and the findings?	Yes	10-19
31. Clarity of major themes	Were major themes clearly presented in the findings?	Yes, in the Tables and text	10-19
32. Clarity of minor themes	Is there a description of diverse cases or discussion of minor themes?	No, this is not described	10-19
