# Supplementary material for: ‘I JUST WANT TO RUN’: how recreational runners perceive and deal with injuries
Source: BMJ Open Sport Exerc Med. 2021 Sep 27;7(3):e001117. doi: 10.1136/bmjsem-2021-001117 (PMC8477337; doi:10.1136/bmjsem-2021-001117)
Supplement: Supplementary data [file bmjsem-2021-001117supp002.pdf]

Appendix 2 Interview guide outlining the structure of the interview and the topics covered.

**Experience & motivation**
How many years have you been running?<br>What is your motivation to run?
**Injury experience**
Have you had a running injury in the past year? If so, what was this injury?<br>What symptoms / complaints did you have?<br>What were the consequences of this injury?
**Injury definition**
What do you generally consider to be an injury?<br>Which aspects define an injury for you?
**Injury-related factors**
What factors do you believe influence / cause a running injury?<br>How do you feel these factors influence / cause a running injury?
**Injury prevention**
Do you feel that running injuries can be prevented? If so, how?<br>Which injury prevention strategies do you use?<br>What have you taken into account when choosing the injury prevention strategies that you use currently used?<br>What motivates you to pursue an injury prevention strategy?
